# The Impact of Lipoproteins on Wound Healing: Topical HDL Therapy Corrects Delayed Wound Healing in Apolipoprotein E Deficient Mice

**DOI:** 10.3390/ph7040419

**Published:** 2014-04-03

**Authors:** Stephanie C. Gordts, Ilayaraja Muthuramu, Ruhul Amin, Frank Jacobs, Bart De Geest

**Affiliations:** Molecular and Vascular Biology, Department of Cardiovascular Sciences, Katholieke Universiteit Leuven, Campus Gasthuisberg, Herestraat 49, bus 911, Leuven 3000, Belgium; E-Mails: stephanie.gordts@med.kuleuven.be (S.C.G.); ilayaraja.muthuramu@med.kuleuven.be (I.M.); rbio5226@gmail.com (R.A.); jacobsfrank@gmail.com (F.J.)

**Keywords:** hypercholesterolemia, high density lipoproteins, LDL receptor gene transfer, wound healing, topical therapy

## Abstract

Chronic non-healing wounds lead to considerable morbidity and mortality. Pleiotropic effects of high density lipoproteins (HDL) may beneficially affect wound healing. The objectives of this murine study were: (1) to investigate the hypothesis that hypercholesterolemia induces impaired wound healing and (2) to study the effect of topical HDL administration in a model of delayed wound healing. A circular full thickness wound was created on the back of each mouse. A silicone splint was used to counteract wound contraction. Coverage of the wound by granulation tissue and by epithelium was quantified every 2 days. Re-epithelialization from day 0 till day 10 was unexpectedly increased by 21.3% (*p* < 0.05) in C57BL/6 low density lipoprotein (LDLr) deficient mice with severe hypercholesterolemia (489 ± 14 mg/dL) compared to C57BL/6 mice and this effect was entirely abrogated following cholesterol lowering adenoviral LDLr gene transfer. In contrast, re-epithelialization in hypercholesterolemic (434 ± 16 mg/dL) C57BL/6 apolipoprotein (apo) E^−/−^ mice was 22.6% (*p* < 0.0001) lower than in C57BL/6 mice. Topical HDL gel administered every 2 days increased re-epithelialization by 25.7% (*p* < 0.01) in apo E^−/−^ mice. In conclusion, topical HDL application is an innovative therapeutic strategy that corrects impaired wound healing in apo E^−/−^ mice.

## 1. Introduction

Decreased high density lipoprotein (HDL) cholesterol levels and elevated non-HDL cholesterol levels are independent risk factors for ischemic cardiovascular diseases [1]. This relationship is very strong for coronary heart disease, but relatively weak for ischemic stroke [1]. However, the medical impact of lipoproteins may extend to disorders unrelated to coronary heart disease and ischemic stroke. Cholesterol is an essential component of mammalian cell membranes and is concentrated in lipid rafts [2]. Clinical and experimental animal studies support a role of cholesterol in heart failure [3,4], neurodegenerative diseases [5], and cancer [6].

HDL is involved in reverse cholesterol transport, but also has anti-oxidative, anti-inflammatory, anti-apoptotic, and endothelial protective properties [7,8]. Furthermore, HDL increases endothelial progenitor cell (EPC) number and function and this may contribute significantly to its atheroprotective properties [9,10,11]. These pleiotropic effects offer perspectives for new areas for HDL therapy that are outside the field of atherosclerosis and vascular biology. The new therapeutic area that is the focus of this study is cutaneous wound healing. Wound healing results from complex interactions between extracellular matrix molecules, soluble mediators, resident skin cells, and infiltrating leukocytes as well as infiltrating EPCs. Rather artificially, wound healing can be divided in an inflammation phase, a phase of tissue formation with accumulation of granulation tissue and re-epithelialization, and finally a phase of tissue remodelling [12]. Chronic non-healing wounds lead to considerable morbidity and mortality. HDL may beneficially affect wound healing by accelerating resolution of inflammation, by enhancing granulation tissue formation involving increased EPC incorporation and increased paracrine effects of EPCs, and by accelerating re-epithelialization.

We have previously developed a new method for therapeutic use of HDL, namely topical HDL administration [13]. One of the main advantages of topical HDL therapy is that the “distribution volume” is small. In addition, the extracellular protein concentration of HDL in humans is 300–400 µg/mL [14,15]. This concentration is approximately 20% of the plasma protein concentration of HDL. Topical HDL application on the adventitial side of vein grafts attenuates vein graft atherosclerosis via increased incorporation of circulating progenitor cells in the endothelium, enhanced endothelial regeneration, and reduced intimal inflammation [13]. The objectives of the current study were: (1) to investigate the hypothesis that elevated plasma cholesterol levels impair wound healing in mice and (2) to study the effect of topical HDL administration in a murine model of delayed wound healing.

## 2. Materials and Methods

### 2.1. Animals

All experimental procedures were approved by the Institutional Animal Care and Research Advisory Committee of the Katholieke Universiteit Leuven (Project: 017/2010-B De Geest-topische HDL). C57BL/6, C57BL/6 apolipoprotein (apo) E deficient, and C57BL/6 low density lipoprotein receptor (LDLr) deficient mice were originally obtained from Taconic (Hudson, NY, USA). All experiments were performed in male mice. C57BL/6 apo E^−/−^ mice were fed normal chow (Sniff Spezialdiäten GMBH, Soest, Germany). C57BL/6 and C57BL/6 LDLr^−/−^ deficient mice received a diet containing 0.2% (w/w) cholesterol and 10% (v/w) coconut oil *ad libitum* starting from the age of 10 weeks or were kept on normal chow. All diets were maintained till the end of the experiment, which occurred at the age of 16.5 weeks.

### 2.2. LDL Receptor Gene Transfer

The generation of the E1E3E4-deleted adenoviral vector AdLDLr has been described before [16,17]. The expression cassette of this vector contains the 1.2 kb DC172 promoter, consisting of an 890 bp human *α_1_-antitrypsin* promoter and two copies of the 160 bp α_1_-*microglobulin* enhancer, upstream of the 5’ untranslated region (UTR) of the human *apo A-I* gene that contains the first intron, and upstream of the 2.6 kb low density lipoprotein receptor (*LDLr)* sequence and 2 copies of the 774 bp *hepatic control region-1*. Large-scale production of recombinant E1E3E4-deleted adenoviral vectors was performed as described previously [18]. In selected experiments, gene transfer with 5 × 10^10^ viral particles was performed three weeks after the start of the diet containing 0.2% (*w/w*) cholesterol and 10% (*v/w*) coconut oil diet in C57BL/6 LDLr^−^^/−^ mice.

### 2.3. HDL Purification

Human lipoproteins were separated from a single plasma pool obtained from five healthy male and five healthy female donors by density gradient ultracentrifugation in a swing-out rotor as described by Jacobs *et al*. [19]. Following HDL (1.063 g/mL < d < 1.21 g/mL) isolation, dialysis was performed against phosphate buffered saline pH 7.4 containing 0.5 mM ethylenediaminetetraacetic acid (EDTA). Subsequently, HDL was concentrated. A bicinchoninic protein assay kit (Pierce Biotechnology Inc., Rockford, IL, USA) was used to quantify protein content in HDL.

### 2.4. Determination of Plasma Cholesterol and of non-HDL and HDL Cholesterol Levels

Mouse lipoproteins were separated by density gradient ultracentrifugation in a swing-out rotor as described before [19]. Fractions were stored at −20 °C until analysis. Total cholesterol in plasma and in isolated non-HDL and HDL fractions was determined with commercially available enzymes (Roche Diagnostics, Basel, Switzerland). Precipath L (Roche Diagnostics) was used as a standard.

### 2.5. Full Thickness Excisional Wound Model

All wounds were created at the age of 15 weeks. Mice were anesthetized with a mixture of ketamin-xylazin (75 mg/kg ketamine, 15 mg/kg xylazin (Rompun)) by intraperitoneal injection. The volume of Rompun (µL) was determined as body weight (g) × 2 + 10. In addition, atropine (0.5–1 mg/kg) was given intraperitoneally to decrease bronchial and salivary secretions. Dorsal and ventral hair was shaved, followed by complete removal of remaining short hairs using a depilatory agent. The animal was then placed on a sterile operation field and the dorsum was disinfected with iodine solution. Thereafter, a pattern for the wound in the middle of the mouse dorsum was traced using a sterilized biopsy punch with a diameter of 5 mm (Instruma, Tienen, Belgium). A full thickness wound extending through the *panniculus carnosus* was made using an iris scissor. A doughnut-shaped splint (Invitrogen, Carlsbad, CA, USA) with an external diameter of 8 mm was placed on the skin so that the wound was centered within the splint. Next, after fixating the splint with a histoacryl tissue adhesive, its position was ensured with 8 6/0 polyamide suture stitches (Ethilon, Johnson & Johnson Medical, New Brunswick, NJ, USA). A semi-occlusive dressing (Tegaderm, 3M, Diegem, Belgium), impermeable to liquids, bacteria, and viruses, was then applied to the wound. Finally, the mice were placed in individual cages, and allowed to fully recover from anesthesia on a heating pad.

In experiments in C57BL/6 apo E^−/−^ mice, the control group was compared with a control gel group and an HDL gel group. In the control gel group, a volume of 80 µL of 20% Pluronic F-127 gel (pH 7.2) (Sigma, Steinheim, Germany) was applied on the wound every two days starting from the day of wound creation. In the HDL gel group, the same volume of 20% Pluronic F-127 gel containing HDL (protein concentration 800 µg/mL) was administered on the wound bed every two days starting from the day of wound creation. Following gel application, a semi-occlusive dressing was applied to cover the wounds and mice were placed on a heating pad till they had completely recovered.

### 2.6. Macroscopic Analysis of the Wounds

The dressing was renewed every two days until day 10 after wound creation. On the day of surgery and every two days thereafter, a digital photograph was taken under isoflurane anesthesia. Photographs were macroscopically analyzed using Image J software (Wayne Rasband, National Institutes of Health, Bethesda, MD, USA). The wound area was measured in two different ways: wound coverage by granulation tissue and wound coverage by newly formed epithelium. This analysis was performed for each wound every two days until day 10.

### 2.7. Statistical Analysis

Continuous variables were summarized by means, standard error of the mean, and sample size, and were compared between two groups by an unpaired t-test using Instat 3 (Graphpad Software, San Diego, CA, USA). If indicated, a transformation (natural logarithm) was created. Continuous parameters between more than two groups were compared by analysis of variance followed by Dunnetts’s multiple comparison post-test *versus* C57BL/6 mice on normal chow. Areas under the re-epithelialization curve from day 0 till 10 were calculated using Prism4 (GraphPad Software). A *p*-value of less than 0.05 was considered statistically significant.

## 3. Results

### 3.1. Severe Hypercholesterolemia in C57BL/6 LDLr^−/−^ Mice Enhances Wound Healing

**Figure 1:**
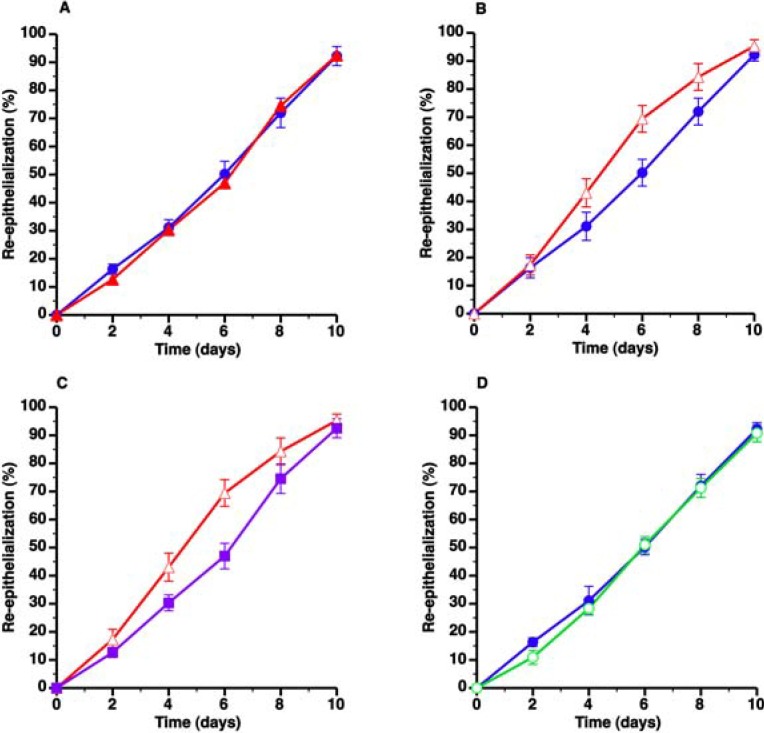
Evaluation of the effect of hypercholesterolemia on wound healing in C57BL/6 LDLr^−/−^ mice. The different panels illustrate the time course of wound coverage by newly formed epithelium expressed as percentage of the original wound surface. Wounds were evaluated every two days from surgical creation of the wound until 10 days later. Panels **A** and **B** represent a comparison of C57BL/6 SC diet mice (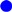) and C57BL/6 LDLr^−/−^ SC diet mice (▲) and C57BL/6 LDLr^−/−^ CC diet mice (Δ), respectively. The effect of AdLDLr gene transfer (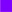) on re-epithelialization in C57BL/6 LDLr^−/−^ CC diet mice is illustrated in Panel **C**. Panel **D** compares wound coverage by newly formed epithelium in C57BL/6 SC diet mice (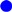) and C57BL/6 CC diet mice (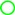). All data represent means ± SEM.

To investigate the hypothesis that elevated plasma cholesterol levels impair wound healing in mice, we first compared wound healing in C57BL/6 mice and in C57BL/6 LDLr^−/−^ mice on standard chow (SC) diet. Plasma cholesterol levels were 2.67-fold (*p* < 0.001) higher in C57BL/6 LDLr^−/−^ mice on SC diet compared to C57BL/6 mice on SC diet (Table 1). Since intergroup differences in wound coverage by granulation tissue were very similar compared to intergroup differences in wound coverage by epithelial tissue, wound coverage data presentation will be mainly focused on re-epithelialization throughout the manuscript. Wound coverage by epithelial tissue was very similar in C57BL/6 LDLr^−/−^ SC diet mice compared to C57BL/6 SC diet mice (Figure 1A, Table 2).

**Table 1:** Total, non-HDL, and HDL cholesterol levels (mg/dl) in murine plasma at the time of wound creation.

	Total cholesterol	Non-HDL cholesterol	HDL cholesterol
C57BL/6 SC diet	71.0 ± 5.1	11.7 ± 1.1	59.2 ± 4.9
C57BL/6 CC diet	78.1 ± 4.1	13.9 ± 1.4	64.2 ± 3.6
C57BL/6 LDLr^−/−^ SC diet	189 ± 10 ***	146 ± 10 ***	42.9 ± 2.9 *
C57BL/6 LDLr^−/−^ CC diet	489 ± 14 ***	419 ± 13 ***	69.8 ± 3.8
C57BL/6 LDLr^−/−^ CC diet AdLDLr	72.3 ± 3.5	27.4 ± 2.7 ***	45 ± 3.4 *
C57BL/6 apo E^−/−^ SC diet	434 ± 16 ***	405 ± 5 ***	29.0 ± 1.4 ***

Data are expressed as means ± SEM (*n* = 10). SC: standard chow. CC: 0.2% cholesterol 10% coconut oil. *p < 0.05; *** *p* < 0.001 *versus* C57BL/6 SC diet.

**Table 2:** Area under the re-epithelialization curve (%. days) from 0 till day 10.

	Number of mice	Area under the curve
C57BL/6 SC diet	10	432 ± 26
C57BL/6 CC diet	12	414 ± 18
C57BL/6 LDLr^−/−^ SC diet	10	424 ± 25
C57BL/6 LDLr^−/−^ CC diet	13	523 ± 31 *
C57BL/6 LDLr^−/−^ CC diet AdLDLr	14	432 ± 27
C57BL/6 apo E^−/−^ SC diet	11	334 ± 11 ***

Data are expressed as means ± SEM. SC: standard chow. CC: 0.2% cholesterol 10% coconut oil. * *p* < 0.05; *** *p* < 0.001 *versus* C57BL/6 SC diet.

Since the degree of hypercholesterolemia in C57BL/6 LDLr^−/−^ SC diet mice is limited, we subsequently analyzed wound healing in C57BL/6 LDLr^−/−^ mice on a diet supplemented with 10% coconut oil and 0.2% cholesterol (CC diet). Plasma cholesterol levels were 6.89-fold (*p* < 0.001) higher in C57BL/6 LDLr^−/−^ mice on CC diet compared to C57BL/6 mice on SC diet (Table 1). Unexpectedly, re-epithelialization from day 0 till day 10 was increased by 21.3% (*p* < 0.05) in C57BL/6 LDLr^−/−^ CC diet mice compared to C57BL/6 mice SC diet mice (Figure 1B, Table 2).

To ascertain that increased re-epithelialization in C57BL/6 LDLr^−/−^ CC diet mice was due to hypercholesterolemia and not due to hypercholesterolemia-independent effects of the CC diet, AdLDLr gene transfer was performed in C57BL/6 LDLr^−/−^ CC diet mice. AdLDLr gene transfer two weeks before wound creation in these mice decreased plasma cholesterol levels 6.76–fold (*p* < 0.0001) (Table 1). Re-epithelialization from day 0 till day 10 in AdLDLr mice was 17.5% (*p* < 0.05) lower compared to C57BL/6 LDLr^−/−^ CC diet mice (Figure 1C, Table 2) and very similar compared to C57BL/6 SC diet mice (Table 2). To further corroborate that the CC diet did not modify wound healing independent of its effects on plasma cholesterol, we compared wound healing in C57BL/6 SC diet mice and in C57BL/6 CC diet mice. Plasma cholesterol levels were not significantly altered by the CC diet in C57BL/6 mice and were 84.0% (*p* < 0.0001) lower in C57BL/6 CC diet mice than in C57BL/6 LDLr^−/−^ CC diet mice (Table 1). Wound coverage by epithelial tissue was very similar in C57BL/6 mice on both diets (Figure 1D, Table 2), but was 20.9% (*p* < 0.01) lower in C57BL/6 CC diet mice than in C57BL/6 LDLr^−/− ^CC diet mice (Table 2). Taken together, severe hypercholesterolemia in C57BL/6 LDLr^−/−^ CC diet mice results in an unexpected improvement of wound healing.

### 3.2. C57BL/6 Apo E^−/−^ Mice Are Characterized by Delayed Wound Healing

**Figure 2:**
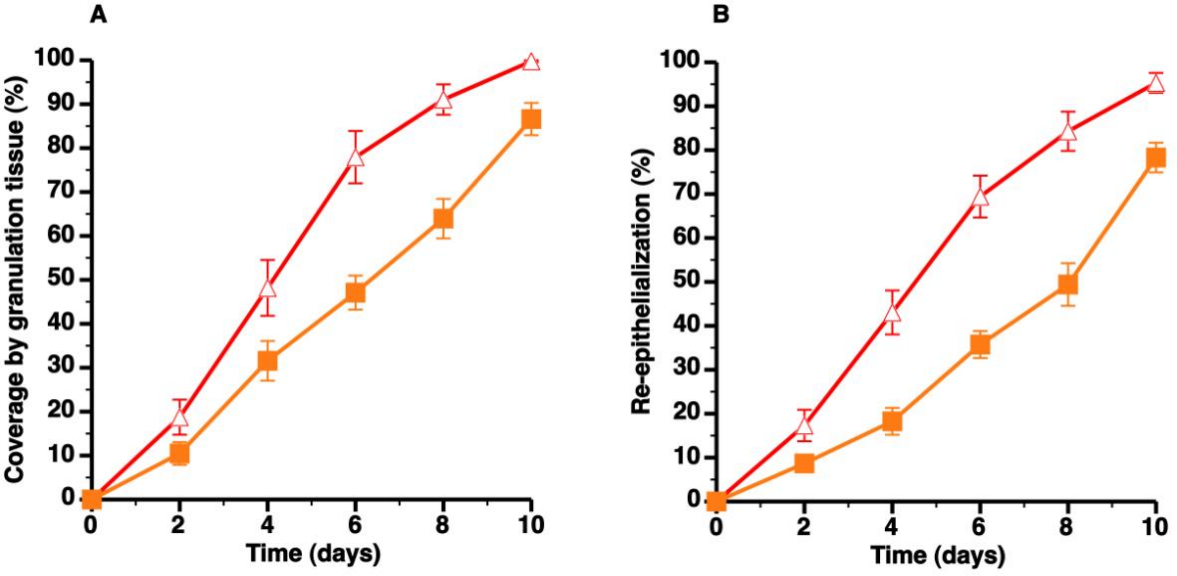
Direct comparison of wound healing in C57BL/6 LDLr^−/−^ CC diet mice (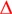) and C57BL/6 apo E^−/−^ SC diet mice (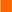). Panels A and B illustrate the time course of wound coverage by granulation tissue and by newly formed epithelium, respectively, both expressed as percentage of the original wound surface. All data represent means ± SEM.

**Figure 3:**
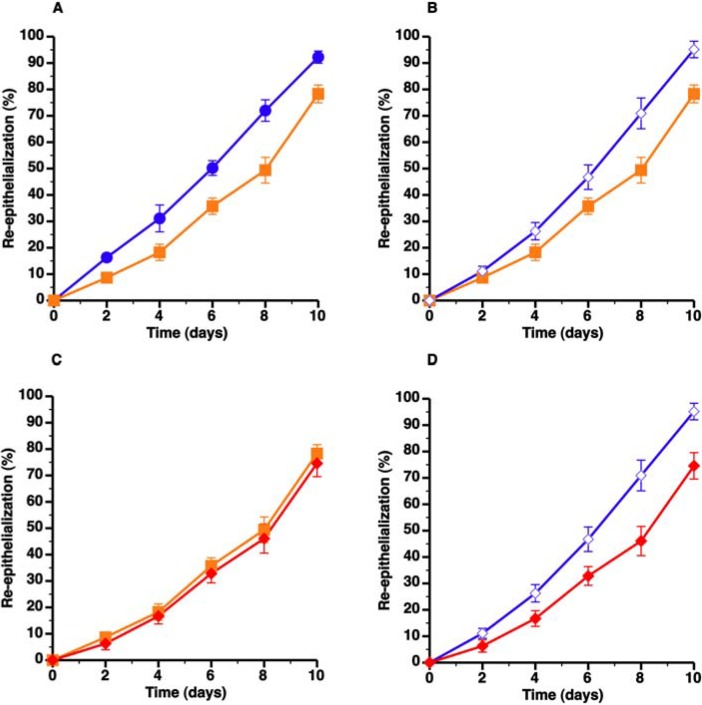
Topical HDL therapy corrects delayed wound healing in C57BL/6 apoE^−/−^ mice. The different panels illustrate the time course of wound coverage by newly formed epithelium expressed as percentage of the original wound surface. Wounds were evaluated every two days from surgical creation of the wound until 10 days later. Panel A illustrates delayed re-epithelialization in C57BL/6 apo E^−/−^ mice (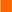) compared to C57BL/6 mice (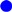) whereas panel B shows that topical HDL therapy (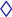) corrects delayed wound healing. Panel C represents a comparison of wound coverage by newly formed epithelium in C57BL/6 apo E^−/−^ control mice (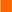) and C57BL/6 apo E^−/−^ mice treated with control pluronic gel (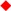). The effect of control Pluronic gel (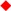) and HDL Pluronic gel (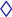) is directly compared in panel D. All data represent means ± SEM.

The striking difference in granulation tissue formation and re-epithelialization between C57BL/6 LDLr^−/−^ CC diet mice and C57BL/6 apo E^−/−^ mice is illustrated in Figure 2A and Figure 2B, respectively. Interestingly, non-HDL cholesterol levels in C57BL/6 apo E^−/−^ mice on SC diet were similar compared to the non-HDL cholesterol concentration in C57BL/6 LDLr^−/−^ CC diet mice (Table 1). However, HDL cholesterol levels were decreased by 51.0% (*p* < 0.0001) and by 58.5% (*p* < 0.0001) compared to C57BL/6 SC diet mice and C57BL/6 LDLr^−/−^ CC diet mice, respectively (Table 1). Re-epithelialization from day 0 till day 10 in C57BL/6 apo E^−/−^ mice was 22.6% (*p* < 0.0001) lower than in C57BL/6 SC diet mice (Figure 3A, Table 2). All in all, C57BL/6 apo E^−/−^ mice constitute a model of delayed wound healing.

### 3.3. Topical HDL Therapy Enhances Wound Healing in C57BL/6 Apo E^−/−^ Mice

**Figure 4:**
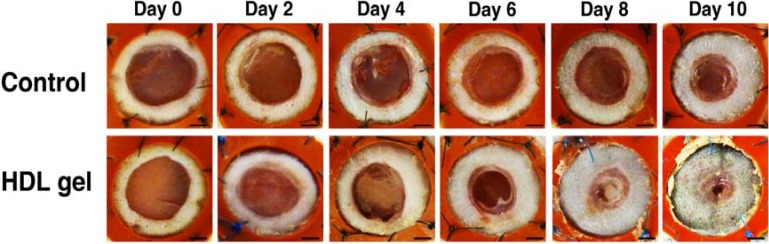
Representative images of the time course of wound healing in C57BL/6 apo E^−/−^ control mice and in C57BL/6 apo E^−/−^ mice treated with topical HDL.

The effect of topical administration of HDL (protein concentration 800 µg/mL; volume 80 µL) formulated in 20% pluronic F-127 gel (pH 7.2) on wound healing was evaluated in C57BL/6 apo E^−/−^ mice. Topical HDL gel administered every 2 days resulted in a 25.7% (*p* < 0.01) increase of re-epithelialization from day 0 till day 10 (Figure 3B, Table 3). Topical gel without HDL did not enhance wound healing (Figure 3C, Table 3). Compared to C57BL/6 apo E^−/−^ mice treated with control gel, re-epithelialization from day 0 till day 10 was increased by 47.8% (*p* < 0.01) in C57BL/6 apo E^−/−^ mice treated with topical HDL gel (Figure 3D, Table 3). Representative images of the time course of wound healing in C57BL/6 apo E^−/−^ control mice and in C57BL/6 apo E^−/−^ mice treated with topical HDL are shown in Figure 4.

**Table 3:** Area under the re-epithelialization curve (%. days) from 0 till day 10.

	Number of mice	Area under the curve
C57BL/6 apo E^−/−^	11	334 ± 11
C57BL/6 apo E^−/−^ control gel	10	284 ± 31
C57BL/6 apo E^−/−^ HDL gel	10	420 ± 28 **^§§^

Data are expressed as means ± SEM. ** *p* < 0.01; *versus* C57BL/6 apo E^−/−^; ^§§^ *p* < 0.01 *versus* C57BL/6 apo E^−/−^ control gel.

## 4. Discussion

The salient findings of the present study are that: (1) severe hypercholesterolemia in C57BL/6 LDLr^−/−^ mice is unexpectedly associated with improved wound healing; (2) in contrast, hypercholesterolemic C57BL/6 apo E^−/−^ mice are characterized by delayed wound healing; and (3) topical HDL therapy corrects delayed wound healing in C57BL/6 apo E^−/−^ mice.

Wound healing in mice occurs predominantly by wound contraction, whereas in humans healing is primarily the result of re-epithelialization and granulation tissue formation [20]. In the excisional wound healing model [21], a circular full thickness wound that extends to the *panniculus carnosus* is created on the back of each mouse. To counteract wound contraction, a silicone splint is fixed around the wound with nylon sutures. Consequently, wound healing in the model used in the current study occurs by granulation tissue formation and re-epithelialization from the border, thereby resembling wound healing in humans.

Our initial hypothesis was that wound healing would be impaired in C57BL/6 LDLr^−/−^ mice. Hypercholesterolemia is associated with EPC dysfunction [22] and decreased EPC number and function has also been demonstrated specifically in C57BL/6 LDLr^−/−^ mice [4,23]. Based on the role of EPCs in granulation tissue formation [24], EPC dysfunction was expected to negatively affect granulation tissue formation. Contrary to these expectations, we observed no effect of moderate hypercholesterolemia and a positive effect of severe hypercholesterolemia on granulation tissue formation and reepithelialisation in C57BL/6 LDLr^−/−^ mice. Since induction of severe hypercholesterolemia in C57BL/6 LDLr^−/−^ mice requires application of a diet containing 0.2% cholesterol and 10% coconut oil (CC diet), improved wound healing in C57BL/6 LDLr^−/−^ CC diet mice might theoretically have been due to dietary effects unrelated to plasma cholesterol levels. However, two sources of evidence unequivocally demonstrate that the improvement of wound healing in C57BL/6 LDLr^−/−^ CC diet mice is directly related to increased plasma cholesterol levels. Firstly, AdLDLr gene transfer attenuates wound healing in C57BL/6 LDLr^−/−^ CC diet mice. Secondly, wound healing is similar in C57BL/6 CC diet mice and in C57BL/6 SC diet mice. Therefore, the improved wound healing in C57BL/6 LDLr^−/−^ CC diet mice suggests that another mechanism overrides the negative impact of hypercholesterolemia on EPC biology. Skin fibroblasts constitute another key cell type involved in granulation tissue formation. It has previously been demonstrated that LDL stimulation of human skin fibroblasts activates p38 mitogen-activated protein kinases (MAPKs) and increases wound-healing capacity of these cells *in vitro* [25]. The effect of LDL on fibroblast p38 MAPK activation and on fibroblast spreading is independent of the LDL receptor [26] and occurs via scavenger receptor class B type I (SR-BI) [27]. SR-BI recognizes a broad variety of lipoproteins, including HDL, LDL, and oxidized LDL [28,29]. We speculate that non-HDL lipoproteins in C57BL/6 LDLr^−/−^ CC diet mice (predominantly intermediate density lipoproteins (IDL) and LDL) may result in positive effects on skin fibroblasts, granulation tissue formation and on wound healing. In contrast, the biochemically highly distinct non-HDL lipoproteins in C57BL/6 apo E^−/−^ mice (predominantly chylomicron remnants and very low density lipoprotein (VLDL) remnants) likely fail to activate p38 MAPK in skin fibroblasts. The impaired granulation tissue formation in these mice may be related to the lower EPC number and to EPC dysfunction [9,30]. However, other factors may contribute to impaired wound healing in apo E deficient mice. Firstly, as indicated by our lipoprotein data, HDL cholesterol levels are reduced in C57BL/6 apo E^−/−^ mice compared to C57BL/6 mice and C57BL/6 LDLr^−/−^ mice. It is possible that low HDL cholesterol levels in C57BL/6 apo E^−/−^ mice play a significant role in impaired wound healing. Secondly, we cannot exclude that apo E itself directly modulates wound healing. Apo E is known to directly modulate oxidation and inflammatory and immune responses [31]. Apo E signalling via the very low VLDL-receptor (VLDL-R) or apo E receptor-2 (apoER2) promotes macrophage conversion from the pro-inflammatory M1 to the anti-inflammatory M2 phenotype [32]. Furthermore, apo E deficiency has been shown to result in a defective clearance of apoptotic bodies and a pro-inflammatory state characterized by increased tumor necrosis factor (TNF)-α and fibrinogen levels in the liver [33]. These effects were not observed in LDLr^−/−^ mice [33]. Based on the direct effects of apo E on the macrophage phenotype and on clearance of apoptotic bodies, apo E deficient mice constitute a model of an altered immune response that is independent of elevated plasma cholesterol levels in these mice. In a model of cryo-injury in the mouse brain, surface size of the wound shrunk more rapidly in the C57BL/6 wild-type mice than in C57BL/6 apo E^−/−^ mice [34]. Interestingly, the lipoprotein system in the brain substantially differs from that in the periphery since the blood brain barrier does not allow passage of lipoproteins. Apo A-I, apo A-IV, apo D, apo E, and apo J are detectable in the brain whereas apo A-II, apo B, and apo C-II are undetectable [35]. Consequently, pro-atherogenic apo B containing lipoproteins do not play any role in the brain. Furthermore, the brain is relatively immunoprivileged. Thus, a role of apo E in wound healing independent of plasma cholesterol levels and immunomodulation is strongly suggested by these brain injury experiments [34]. A similar action of apo E may potentiate cutaneous wound healing. Interestingly, apo E is expressed in basal epithelial cells of the epidermis [36]. Taken together, there are converging lines of evidence that apo E may modulate wound healing independent of its effects on lipoprotein metabolism.

A critical finding in the current study is that topical HDL therapy potently improved wound healing in C57BL/6 apo E^−/−^ mice. Human HDL was formulated in 20% pluronic F-127 gel (pH 7.2). Pluronic F-127 is a biocompatible and non-toxic substance, and is characterized by thermoreversible gel formation at temperatures above 21 °C [37]. HDL may beneficially affect wound healing by its anti-inflammatory and immunomodulatory effects [38,39], by enhancing granulation tissue formation involving increased EPC incorporation and increased paracrine effects of EPCs, but also by accelerating re-epithelialization. Not only skin fibroblasts but also keratinocytes express the HDL receptor SR-BI [40]. Furthermore, SR-BI expression is up-regulated in dividing keratinocytes [40].

A few limitations of the current study should be acknowledged. A first limitation of this study is that healing of a surgically created wound was investigated. Healing of an acute wound may differ in several aspects from healing of a chronic wound for which no experimental animal model is available. A second limitation is that we tested only one dose of HDL. Thirdly, the study was restricted to male mice. Wound healing differs between both sexes [41,42,43,44]. Finally, further mechanistic studies are required to prove direct effects of apo E on wound healing and to evaluate which mechanisms underlie the inhibitory effect of AdLDLr gene transfer on cutaneous wound healing in C57BL/6 LDLr^−/−^ CC diet mice.

In summary, the effect of hypercholesterolemia on cutaneous wound healing is highly dependent on the specific murine strain. The opposing effects of hypercholesterolemia on wound healing in C57BL/6 LDLr^−/−^ mice and C57BL/6 apo E^−/−^ mice may be related to distinct effects of pro-atherogenic lipoproteins on skin fibroblasts and granulation tissue formation, to differences of HDL cholesterol levels in these models, and to direct effects of apo E on wound healing. Topical HDL application is an innovative therapeutic strategy that corrects impaired wound healing in apo E deficient mice. Further preclinical studies are required to evaluate the robustness of this strategy in other models of delayed wound healing.
